# An Investigation of Small GTPases in relation to Liver Tumorigenesis Using Traditional Chinese Medicine

**DOI:** 10.1155/2014/428210

**Published:** 2014-06-19

**Authors:** Tzu-Chieh Hung, Wen-Yuan Lee, Kuen-Bao Chen, Yueh-Chiu Chan, Calvin Yu-Chian Chen

**Affiliations:** ^1^Department of Biomedical Informatics, Asia University, Taichung 41354, Taiwan; ^2^School of Medicine, College of Medicine, China Medical University, Taichung 40402, Taiwan; ^3^Department of Neurosurgery, China Medical University Hospital, No. 2 Yude Road, North District, Taichung 40447, Taiwan; ^4^Department of Anesthesiology, China Medical University Hospital, Taichung 40447, Taiwan; ^5^Research Center for Chinese Medicine & Acupuncture, China Medical University, Taichung 40402, Taiwan; ^6^Human Genetic Center, Department of Medical Research, China Medical University Hospital, Taichung 40447, Taiwan

## Abstract

Recently, an important topic of liver tumorigenesis had been published in 2013. In this report, Ras and Rho had defined the relation of liver tumorigenesis. The traditional Chinese medicine (TCM) database has been screened for molecular compounds by simulating molecular docking and molecular dynamics to regulate Ras and liver tumorigenesis. Saussureamine C, S-allylmercaptocysteine, and Tryptophan are selected based on the highest docking score than other TCM compounds. The molecular dynamics are helpful in the analysis and detection of protein-ligand interactions. Based on the docking poses, hydrophobic interactions, and hydrogen bond variations, this research surmises are the main regions of important amino acids in Ras. In addition to the detection of TCM compound efficacy, we suggest Saussureamine C is better than the others for protein-ligand interaction.

## 1. Introduction

Recently, an important topic of liver tumorigenesis had been published in 2013. In this report, Ras and Rho had defined the relation of liver tumorigenesis. Ras and Rho are an important target for liver tumorigenesis [1].

The liver tumorigenesis (or means hepatic carcinoma) is a serious disease in the world, especially in Asia. The main cause of liver tumorigenesis is virus (HBV or HCV) and abnormal eating and habits (smoke, drink, barbecue, raw food, fatigue, and staying up late). The cumulative burden decreases immunity and then makes liver tumorigenesis. The most cases of hepatic carcinoma are found too late to treat and there is no efficient drug that could prevent disease progression which then causes the patient's death.

RAS (also called GTPase Kras) and Rho small GTPases are key molecular switches that control cell dynamics, cell growth, and tissue development through the signaling pathways. Previous references reported the inhibition of Ras for cancer treatment is viable [2–4]. The issue also indicates the activation of RhoA can abate Kras-induced liver tumorigenesis [1]. Thus, the activation of RhoA and the inhibition of Kras may be a well inspire for hepatic carcinoma treatment.

Computer-aided drug design (CADD) is an* in silico* simulation technique to screen for novel compounds by their structure and to predict the biological activity of drug candidates. In comparison with traditional drug design, CADD has the advantages of both greater speed and lower cost. The two major application areas of CADD are structure-based drug design and ligand-based drug design. We used CADD to investigate the basics of molecular simulation in drug design centered on structure-based drug design and molecular dynamics [5–8].

Recently, there are more attentions on personalized medicine and biomedicine [9]; then this knowledge could analyze the mutation [10], and cause for special disease [11]. Traditional Chinese medicine (TCM) is a model of personalized medicine. TCM has an important role in Asia, especially in China, Taiwan, Korea, and Japan. The TCM Database@Taiwan (http://tcm.cmu.edu.tw/) [12] is the largest traditional Chinese medicine database in the world. This database contains 2D chemical structures, 3D chemical structures, bioactivity, and molecular information for over 61,000 compounds of traditional Chinese medicinal herbs. Since 2011, there have been successful discoveries of novel lead compounds from the TCM Database@Taiwan for cancer treatment [13–16], pain relief [6], and antivirals [17–19]. With the assistance of the application system of the website [20] and the cloud computing platform [21], the TCM Database@Taiwan could be valuable for TCM application and drug design.

In this study, we screen a possible lead compound against liver tumorigenesis from the TCM Database@Taiwan. We use the computational techniques of docking screening to select ligands. Finally, we apply molecular dynamics (MD) simulation to investigate variations from protein-ligand interactions that may contribute to the evaluation of the effect on Ras inhibition.

## 2. Materials and Methods

### 2.1. Data Set

In this research, the molecular simulations platform was using Accelrys Discovery Studio 2.5 (DS 2.5). Amount of 61,000 TCM compounds was downloaded from the TCM database (http://tcm.cmu.edu.tw/); then human Ras protein (PDB ID: 4EPV) crystal structure was obtained from RCSB Protein Data Bank [4].

### 2.2. Disorder Protein Detection

The disorder plays an important role in drug design; thus we take the sequence generated from Uniprot to predict the disorder region by the Database of Protein Disorder (DisProt: http://www.disprot.org/). The result of prediction was helpful to decide the character of the docking site and the efficacy of the drug [22].

Taking a comparison of the disorder regions and the docking sites could assess the protein-ligand interaction and drug efficacy.

### 2.3. Molecular Docking

The docking simulation applied on LigandFit module [23] a receptor-rigid docking algorithm program in Discovery Studio 2.5 (DS 2.5) to dock TCM compounds to Ras in the CHARMm force field [24]. The docking site of Ras was identified by the research [3, 4]. After docking, the top three docking scores of the TCM compounds were selected to process the analysis of the hydrophobic interactions by Ligplot [25, 26].

### 2.4. Molecular Dynamics Simulation

These ligands from candidate complex must be reprepared based on the reference force field [27] of GROMACS 4.5.5 [28] by using SwissParam (http://swissparam.ch/) [29] before applying MD simulation. The Ras protein combines with ligands as the complex goes into the buffer/solution simulation box. With a minimum distance of 1.2 Å from the complex, the cubic box was solvated with the TIP3P water model in which sodium and chloride ion were added to neutralize complex charges. The minimization for complex is on the basis of the steepest descent method for 5,000 steps; then the last structure was transferred to MD simulation. The calculations for electrostatic interactions were based on the particle-mesh Ewald (PME) method [30] in which each time step was 2 fs and the numbers of steps were 5,000,000 times. The 100 ps constant temperature (PER ensemble) for equilibration was on the basis of the Berendsen weak thermal coupling method. The total simulation time of MD was 10,000 ps. MD trajectories, RMSD, energy variations, and eigenvector were analyzed using a series of protocols in Gromacs.

## 3. Results and Discussion

### 3.1. The Detection of Disorder Protein

**Figure 1:**
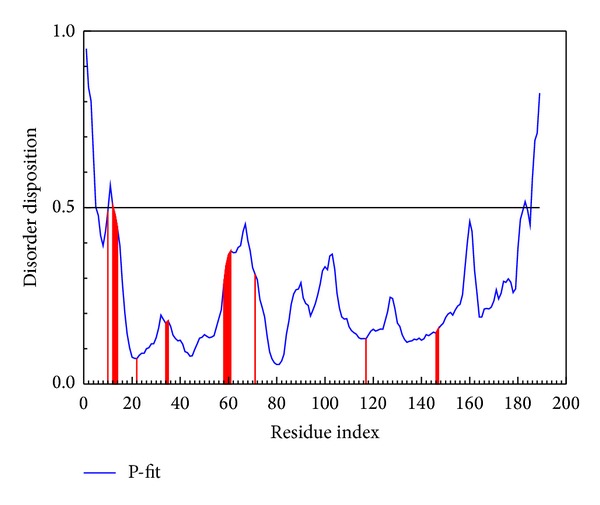
The disorder and binding site detection. The blue curve in the top figure is the disorder disposition of each amino acid and the red lines are the residues of the important amino acids.

The disorder protein is an unstructured protein. For this character, while the docking site is a disorder region, the compounds dock to protein and stabilize the complex with difficultly. In some cited references, [8, 22] also indicate that the disorder region does not consist of any defined domain; thus the drug that can interact with disorder region may have lower side effect than the widespread domain. The disorder region can be defined better as a hard work for drug design than as a bad docking site for selection in our known. The disorder regions are defined as having a disposition of over than 0.5 (Figure 1). In this result, the desposition of important amino acids is less than the threshold; then the ligand docks to the selected site may be appropriate and our results have a weaker effect from disorder protein.

### 3.2. Molecular Docking

Ranking the result of molecular docking by docking score, the top three TCM compounds can be selected (Table 1). These TCM compounds are Saussureamine C, S-allylmercaptocysteine, and Tryptophan extract from the TCM herbs* Saussurea lappa* Clarke,* Allium sativum*, and* Isatis indigotica* Fort., respectively. The top compound, Saussureamine C, is defined as an antiulcer compound [31] and the herb* Saussurea lappa* Clarke can prevent breast cancer cell migration [32], treat heart disease [33, 34], have antihepatotoxic activity [35], and express the killing function of cytotoxic T lymphocytes [36]. The second ranked herb,* Allium sativum*, can antimicrobially [37–39] ameliorate tamoxifen-induced liver injury [40] and with the compound S-allylmercaptocysteine is hepatoprotective and inhibits cancer [41–45]. The third ranked compound, Tryptophan from the herb* Isatis indigotica* Fort., can prevent acute fatal liver failure [46] and make the activation of the differentiation potential for liver progenitor cells [47]. As reported in these literatures, most of these compounds can have an effect on immunity and antihepatotoxic activity. For the above reasons, we suggest that the selected compounds can have effect on Ras.

**Figure 2:**
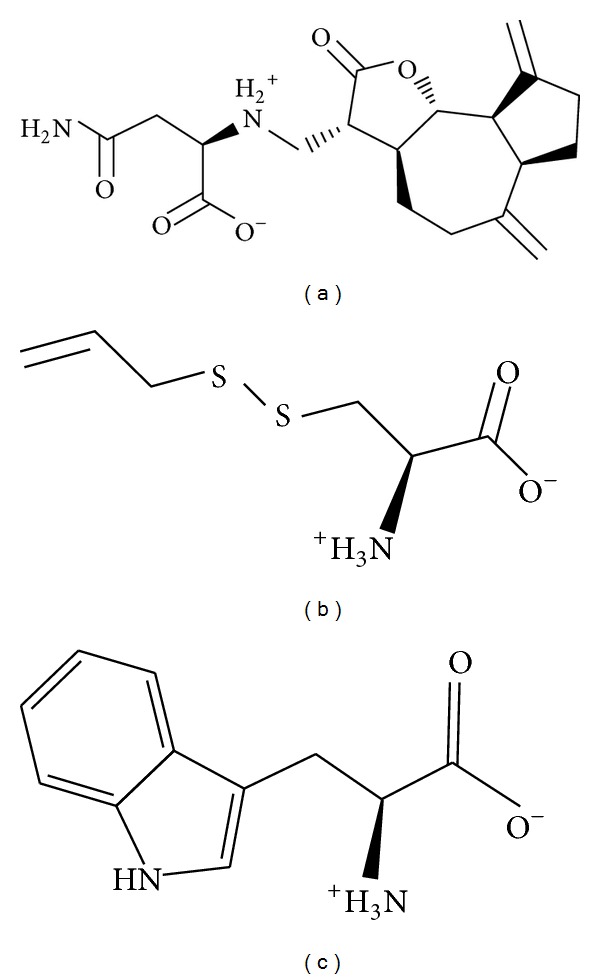
The structure of control and candidate TCM compounds. (a) Saussureamine C, (b) S-allylmercaptocysteine, and (c) Tryptophan.

**Figure 3:**
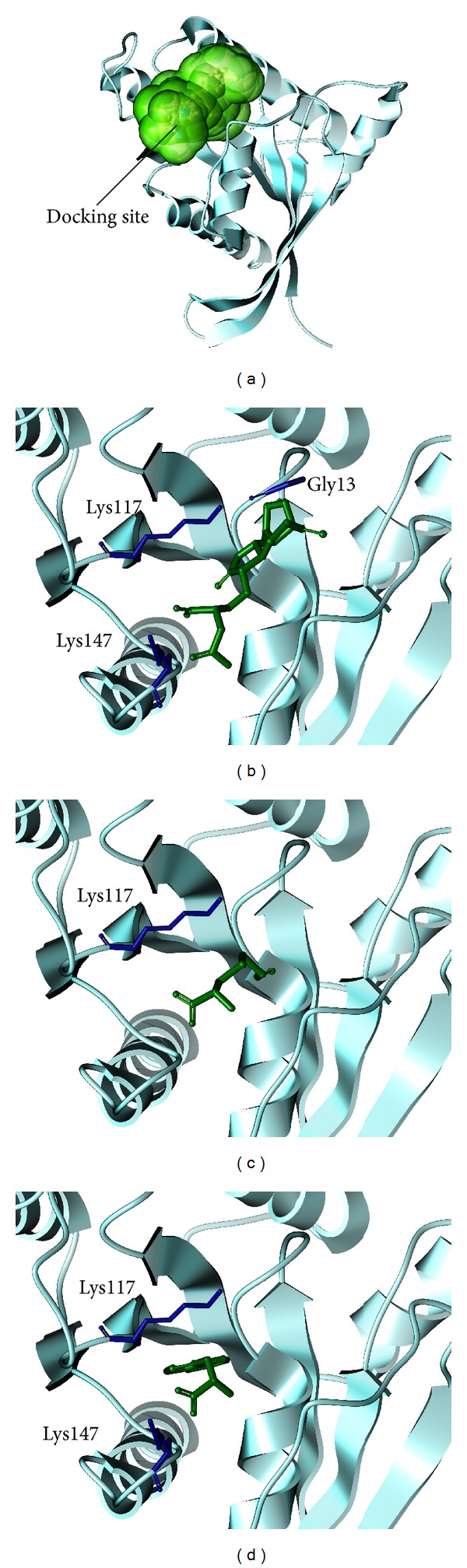
The docking poses of ligands. (a) The crystal structure of Ras and Rho and the docking site: (b) Saussureamine C, (c) S-allylmercaptocysteine, and (d) Tryptophan.

The structure of the candidate compounds is selected after screening from the TCM database (Figure 2); then the docking poses sign the docking site and the important amino acid on the neighbors by ligands (Figure 3). From this result, we observe Lys117 is defined as the amino acids that can interact with all ligands; therefore we suggest these amino acids may play important roles in a Ras target function.

**Figure 4:**
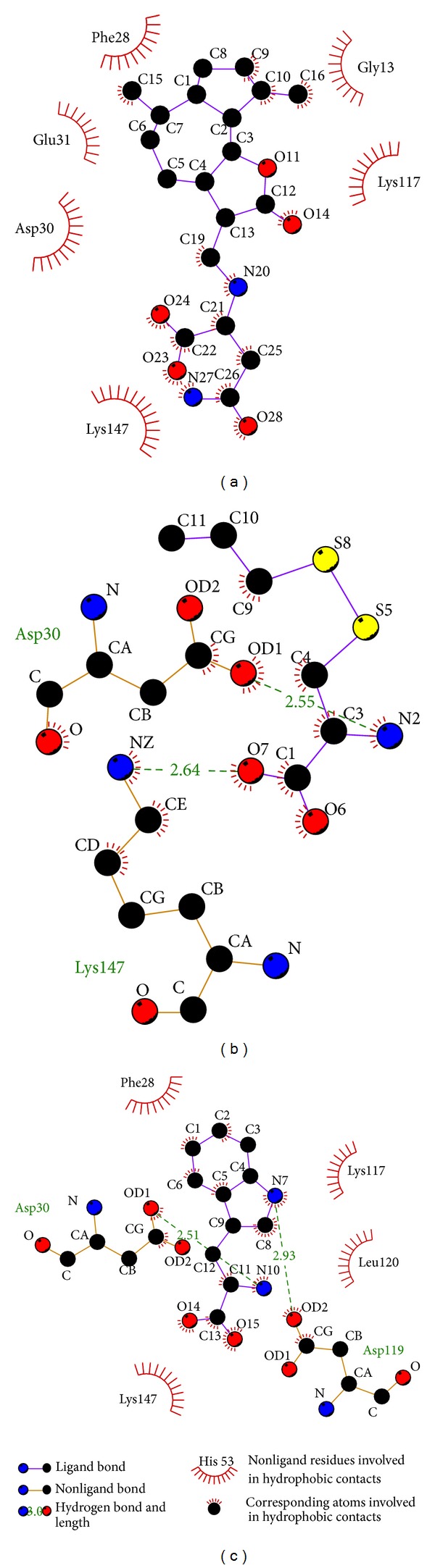
Ligplot illustrates the protein-ligand interactions. (a) Saussureamine C, (b) S-allylmercaptocysteine, and (c) Tryptophan. The deep red color of the hydrophobic interactions indicates a high frequency in all ligand interactions.

The hydrophobic interaction can be analyzed by ligplot (Figure 4). This result shows that the amino acids Asp30 and Lys147 can have interactions with the ligands through hydrophobic interactions or hydrogen bonds. These amino acids might be as important amino while the selected compounds have an effect on Ras.

### 3.3. Molecular Dynamics Simulation

**Figure 5:**
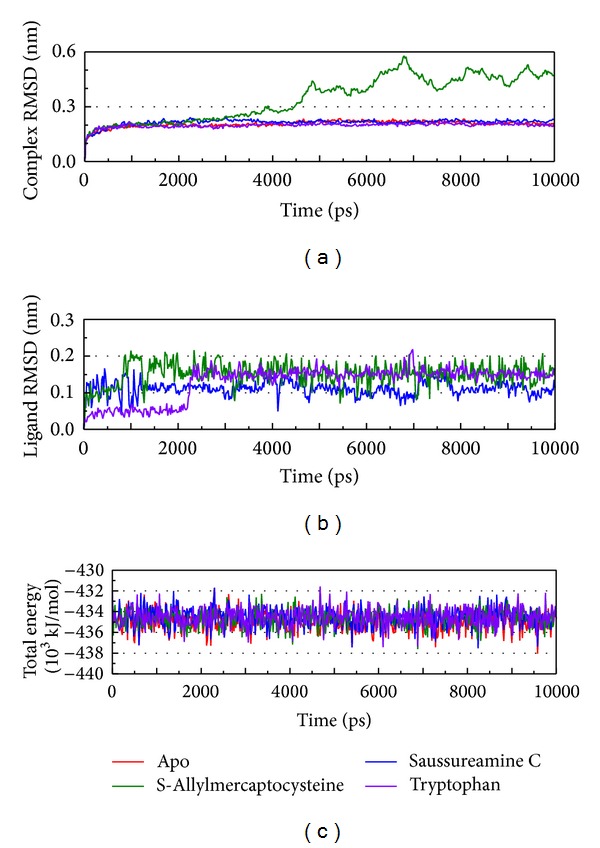
Measures of the MD trajectories. (a) Complex RMSD, (b) ligand RMSD, and (c) the total energy.

The RMSD and total energy of a complex during MD simulation were recorded (Figure 5). The total energy is in the range of −430~−438 ∗10^3^ kJ/mol and tends to −434.5 ∗ 10^3^ kJ/mol. The amplitude is gentle; then we suggest the interaction for Ras and compounds are finished (or mean balance). Besides S-allylmercaptocysteine, the other two compounds do not have large variation. The smaller variation of ligand RMSD indicates the complex will have less interaction and tend to balance. The Saussureamine C has the largest complex RMSD after 3 ns that is presented; the ligand moves away from the docking site recorded in MD.

**Figure 6:**
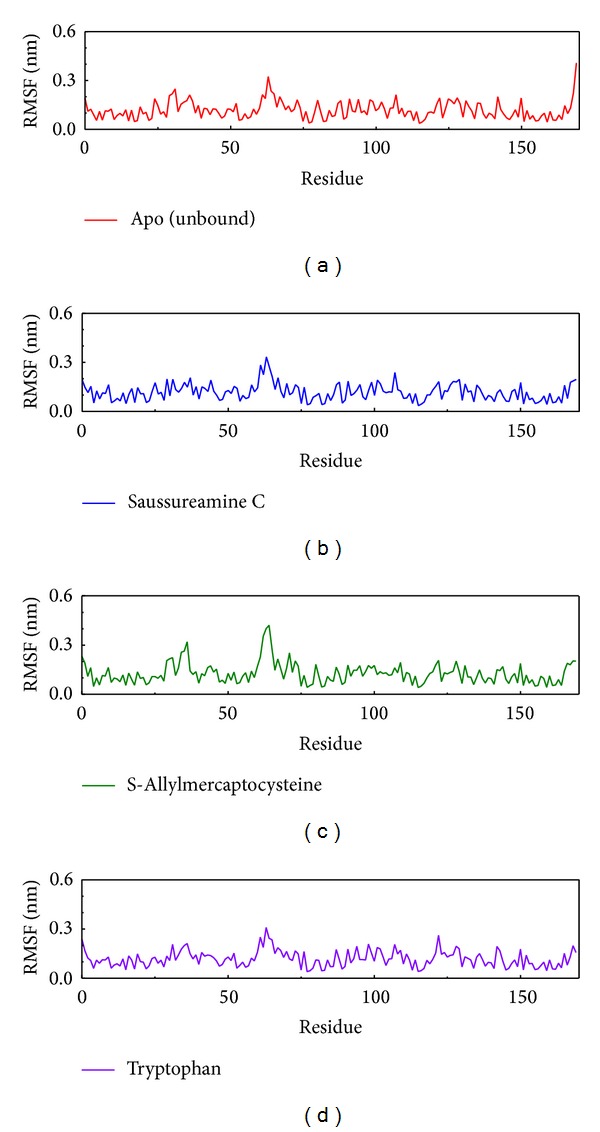
The variation of RMSD focus on residue of protein.

We analyze RMSF which is the RMSD focus on each residue to detect the variation of protein while with ligand interaction (Figure 6). According to this result, we can find the pick sites of residues are similar between Apo form and protein-ligand interaction. The variation of these sites are different caused from ligand interaction.

**Figure 7:**
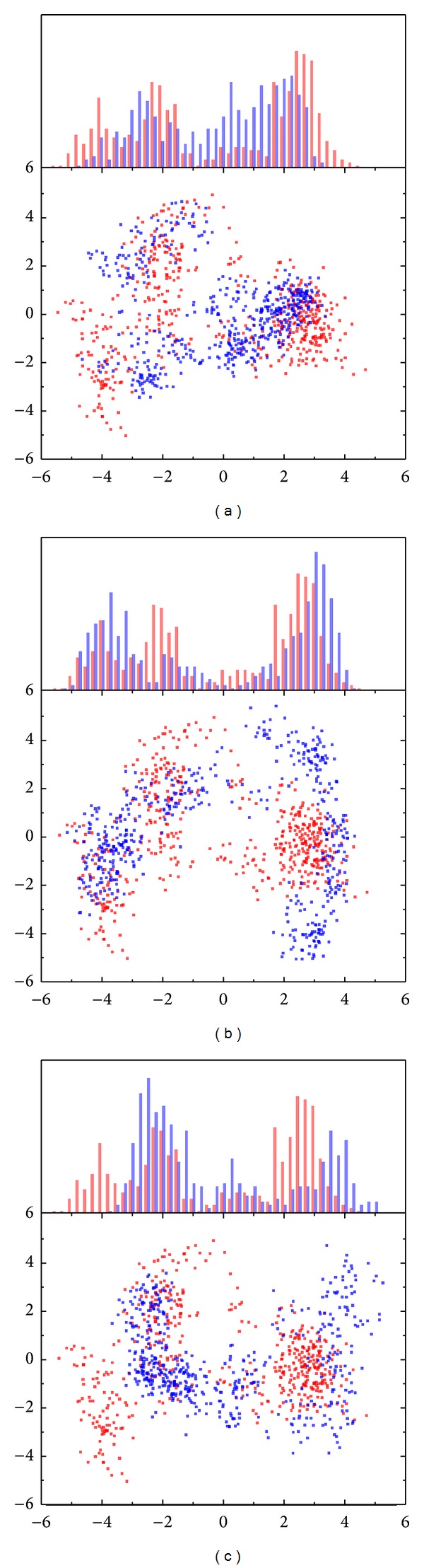
The PCA-eigenvector between ligand and unbound protein. The projection to the first two PCA-eigenvectors as *x* and *y* axes based on the backbone of Ras. The comparison of eigenvector between apo and (a) Saussureamine C, (b) S-allylmercaptocysteine, and (c) Tryptophan.

The reference-identified eigenvector was used to represent the protein variation [48]. The first two PCA (principal component analysis) eigenvectors would become the *x*- and *y*-axes and make the comparison with apo (unbound protein) find protein variation of the first main character of protein (Figure 7). Up in the figure is the first eigenvectors diffusion between apo and complex. The following figure is matrix from first two eigenvectors. After the comparison, we find complex with S-allylmercaptocysteine is similar to apo then different from other compounds. That is because this complex lost the interaction from compound after 30000 ps; then protein interaction was weaker than others. From this result, eigenvector could help in interaction evolution.

**Figure 8:**
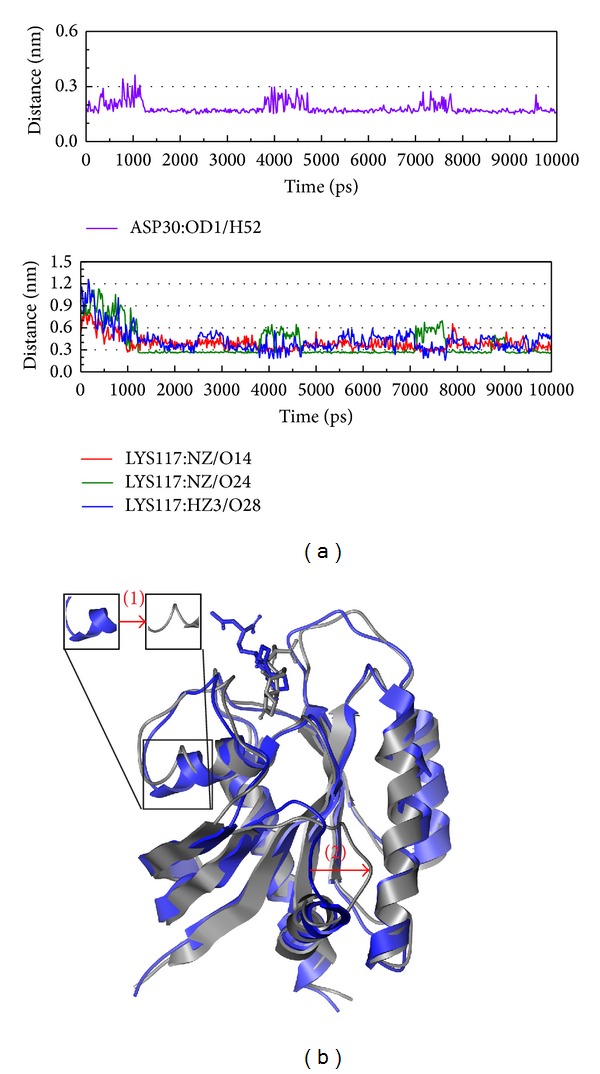
The variation of Saussureamine C and Ras complex in MD simulation. (a) H-bond variation and (b) structure variation. The (1)-(2) red color indicates the difference through MD.

**Figure 9:**
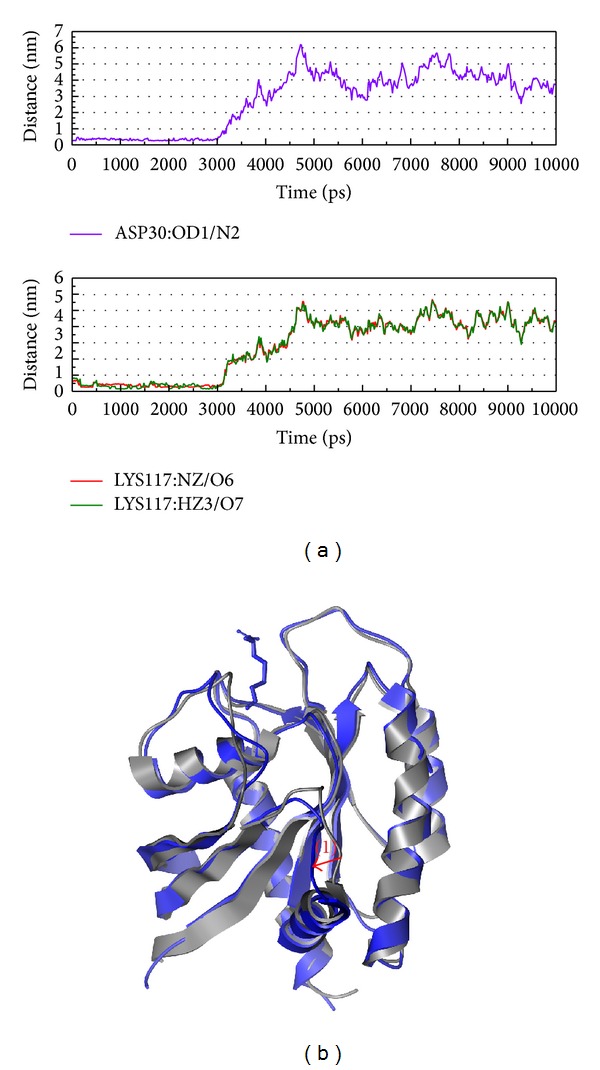
The variation of S-allylmercaptocysteine and Ras complex in MD simulation. (a) H-bond variation and (b) structure variation. The red color indicates the difference through MD.

**Figure 10:**
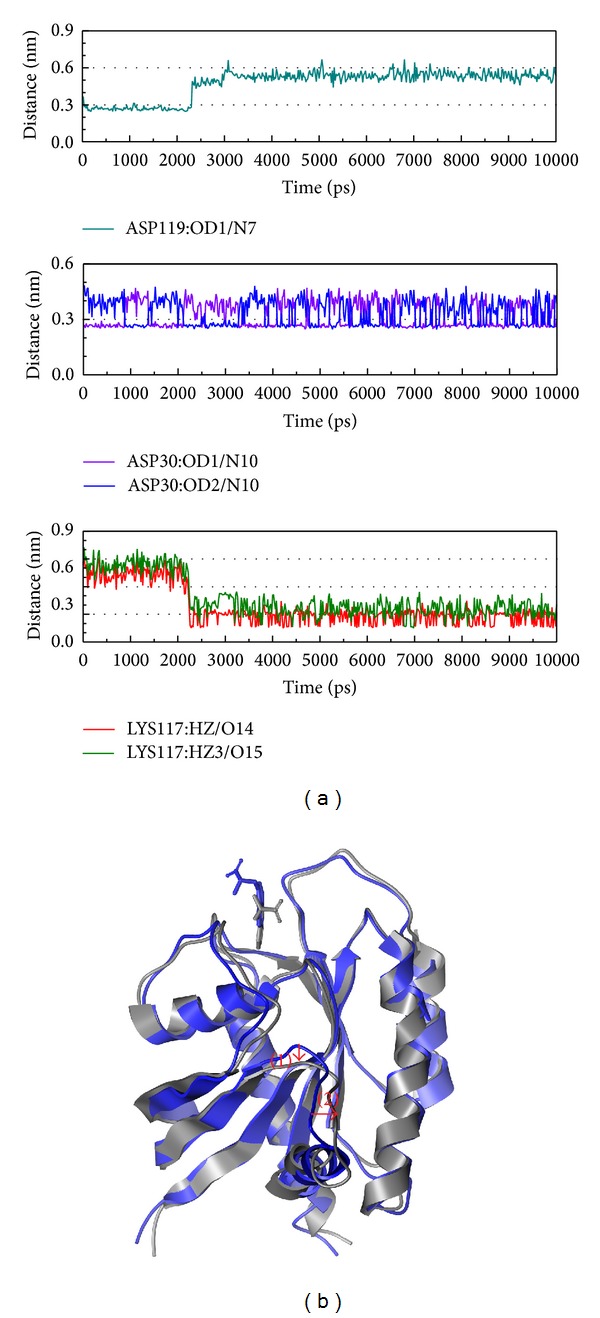
The variation of Tryptophan and Ras complex in MD simulation. (a) H-bond variation and (b) structure variation. The (1)-(2) red color indicates the difference through MD.

After the structure variation discussion is based on eigenvector, we should focus on the structure variation during protein-ligand interaction (Figures 8 to 10). In Figure 8(a), there is high percentage occupancy in H bond from Asp30 and Lys117 after MD 1 ns that present these two amino acids may have the function to inhibit the Ras.  Figure 8(b) shows the variation between MD 0 ns and 10 ns. From this result, we find the protein has two obvious changes on both position and even composition; then we suggest the structure might make Ras lose the function and be inhibited.

Although the complex of S-allylmercaptocysteine and Ras has higher docking score and a lot of relation references, this complex is not stable. The Asp30 and Lys117 also present the important interaction in Figure 9(a) and some region of the structure has variation in Figure 9(b). From these results, we suggest S-allylmercaptocysteine may have better efficacy from the other protein than Ras to control the liver tumorigenesis and the S-allylmercaptocysteine may have a short term effect on Ras.

The Tryptophan complex interactions were recorded (Figure 10). In Figure 10(a), besides Asp30 and Lys117, the Asp119 plays the important role on interaction before 2 ns. There are some position variations caused from Tryptophan interaction in Figure 10(b).

From these variations found, we suggest Saussureamine C could make the largest force on the inhibition of Ras.

**Figure 11:**
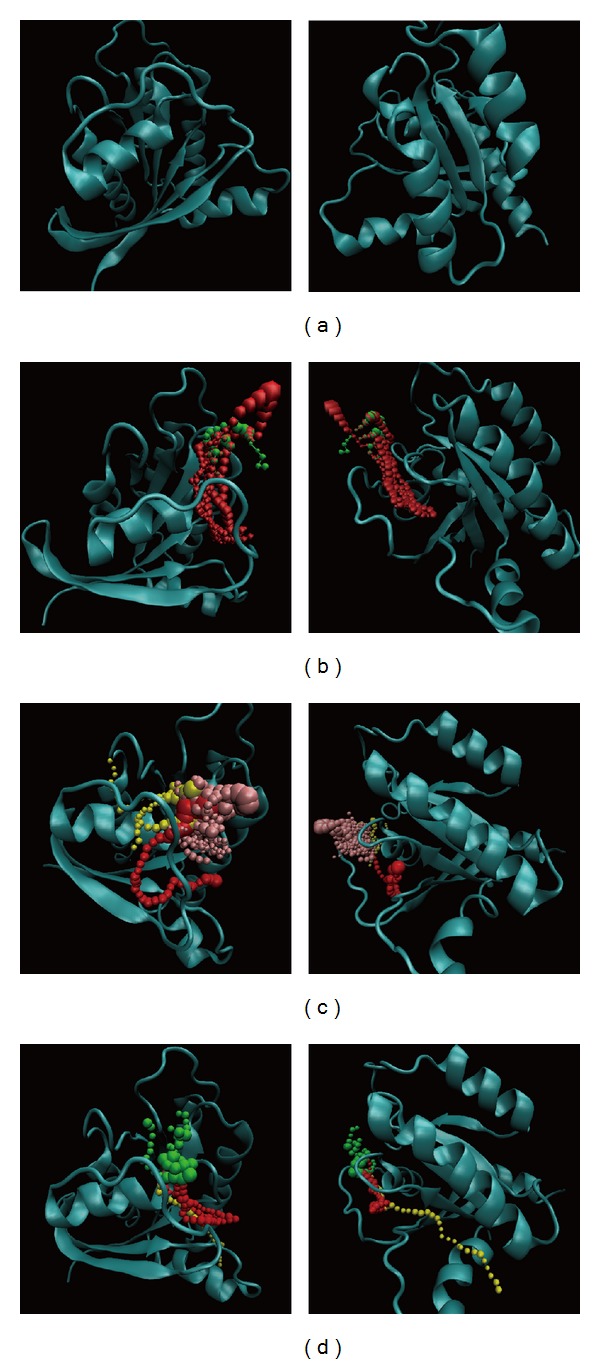
The pathway of Ras for compounds. (a) Unbound protein, (b) Saussureamine C, (c) S-allylmercaptocysteine, and (d) Tryptophan.

Finally, the pathway definition which is based on the calculation of caver 3.0 to determine the interpath protein path during MD simulation [49] could help find out the ligand moving and the pole provided from protein after interaction (Figure 11). In these pathways, we could find the compounds moving around the docking site then into protein. We suggest the inhibition site of Ras might be around the docking site and from the interaction the Ras will be inhibited.

## 4. Conclusion

Based on above discussion, we can find the top three TCM compounds Saussureamine C, S-allylmercaptocysteine, and Tryptophan can have an effect on Ras against liver tumorigenesis. Asp30 and Lys117 might present their effects on Ras when compounds bind or interact with protein. Even S-allylmercaptocysteine has a lot of references to identify the efficacy on this disease but the result of simulation indicates this compound may have more effect on the other protein then represent the regulation. Finally, according to the discussion from docking, interaction, and variation, we suggest Saussureamine C might be a best compound to inhibit Ras against liver tumorigenesis.

## Figures and Tables

**Table 1:** Scoring functions of the top three compounds and the inhibition of Ras.

Compounds	Herbs	-PLP1	-PLP2	Dock score
Saussureamine C	*Saussurea lappa *Clarke	35.6	35.37	196.626
S-Allylmercaptocysteine	*Allium sativum *	21.01	27.18	185.706
Tryptophan	*Isatis indigotica *Fort.	41.19	44.91	184.146
